# Finding Flourishing in Neurology: Increasing Outpatient Exposure, Enhancing Simulated Clinics, and Optimizing Fellowship Training in Epilepsy

**DOI:** 10.1212/NE9.0000000000200038

**Published:** 2022-11-29

**Authors:** Roy Strowd

**Affiliations:** From the Department of Neurology, Wake Forest University School of Medicine, Winston Salem, NC.

In this second issue of *Neurology*® *Education*, readers will find articles grouped in 3 sections. Each section addresses a timely question for educators in clinical neuroscience, including the following: (1) how can simulation be used to optimally teach or assess learners, (2) how can residency training adapt to increase the breadth and depth of outpatient exposure, and (3) what are important educational gaps that need to be addressed for epilepsy fellows and for well-being in neurology broadly?

At its inception over a century ago, training in neurology was exclusively an outpatient experience. Clinical neurology was essentially taught through weekly clinics in the ambulatory setting.^1^ In the early 20th century, a transition began toward more inpatient exposure because training models shifted from individual apprenticeship to board certification and standardized internships in hospitals. Academic departments were built around inpatient wards and training followed suite. The need to increase outpatient exposure is not new. As early as the 1930s, clinicians and educators called for increasing outpatient encounters for trainees.^2^ Multiple reports in the 1990s showed that residents were spending less than 25% of their training in outpatient clinics despite most neurologists practicing more than 80% or more in the ambulatory setting.^3-5^ In fact, 57% of neurologists at that time even favored adding an extra year of residency to accommodate more outpatient experiences.^6^

What has happened in the last 30 years? Despite expanded requirements for outpatient training by residency review committees and increased numbers of outpatient rotations in residency, many of the same challenges remain.

## Preparing Residents for Outpatient Neurologic Practice

The first 2 articles in this issue address solutions to expand outpatient exposure and enhance training in ambulatory neurology and neuropalliative care. The first article from Roy et al. explores how to implement an X + Y scheduling model to increase outpatient exposure in neurology residency training.^7^ The authors implemented 4 + 2 scheduling where residents complete 2 weeks of dedicated outpatient clinics for every 4 weeks of inpatient service. This has become an increasingly popular scheduling approach in internal medicine and pediatrics.^8,9^ Compared with the traditional block training model, the 4 + 2 schedule resulted in a dramatic increase in outpatient clinic time from 4 to 15 weeks per year; patient handoffs were reduced; no change in work hours was reported; and residency in-service training examination scores improved for outpatient topics. Residents were not only more satisfied with the X + Y model but also reported improved well-being.

Is this feasible in all neurology programs? In a prior survey of internal medicine programs, 44% reported using an X + Y schedule.^10^ Most of the programs (53%) tended to be larger, but 23% were medium-sized, and 24% were considered small programs. The most common schedule was a 4 + 1 model. Most of the programs selected the X + Y approach to reduce resident stress, improve resident focus on their current rotation, avoid duty hour violations, and increase outpatient exposure. These data support efforts to consider X + Y scheduling in neurology and provide a perspective on benefits to residents and programs. Adaptations may be needed in neurology residency programs, but the data from Roy et al. provide an important road map for those considering the X + Y approach.

In the second article in this issue, authors from the University of Washington describe an innovative curriculum for enhancing neuropalliative care training for neurology residents.^11^ They used an openly available evidence-based online toolset to meet the Accreditation Council for Graduate Medical Education proficiency milestones in palliative care. The authors applied a transformative learning theory framework to understand how an experiential, workshop-based curriculum can get learners out of their comfort zone, question their ways of thinking, and develop new habits and behaviors. The lessons learned from this group could help other residency and fellowship directors address neuropalliative care training requirements for their learners.

## Simulation in Neurologic Education

Simulation has deep roots in neurology. Dr. Howard Barrows, a neurologist at the University of Southern California in Los Angeles, is widely credited as having blazed the first trail for using simulated patients in a problem-based learning curriculum in the 1960s. His work was met with animosity from medical faculty who challenged whether students could learn from “fake” patients.^12^ Decades later, simulation has become commonplace in medical education and enjoys widespread use in training students, residents, fellows, faculty, interdisciplinary, and even virtual teams. In some respects, neurology has been left behind by colleagues in surgery, anesthesiology, and other specialties who have expanded use of simulation broadly.^12^

The second set of articles and editorial by Dr. Casey Albin provide perspectives on the current state, areas of need, and 2 innovative approaches to using simulation in neurology.^13,14^ New avenues exist to use simulation for competency-based assessment, just-in-time training for procedures and ultra-rare diagnostic investigations, and standardizing training across sites, campuses, and geographic regions. The 2 approaches described by Tchopev et al. and Pergakis et al. will be useful for residency program directors and other educators implementing simulation to assess resident performance reliably and systematically.

## Identifying Educational Gaps to Improve Fellowship Training and Promote Flourishing

The final 2 articles in this issue provide important needs assessment data from educators in the United States and Europe. The first of these articles explores gaps in surgical epilepsy training for clinical neurophysiology fellows in the United States.^15^ This survey-based study highlights 3 existing educational gaps including the following: (1) variability in surgical epilepsy training exposure exists, (2) fellows were most involved in preoperative planning with varied participation in perioperative and postoperative management, and (3) direct observation of fellow competence in surgical management is rare. These gaps can be the source for new innovations to identify, develop, and evaluate methods to assess fellow competence, a road map that could be used by educators in many neurologic fellowship and not just epilepsy.

The second of these articles explores burnout rates as well as factors that contribute to burnout, well-being, and work-related distress in residents, research fellows, and junior faculty in European training programs.^16^ Much has been written in the past decade about burnout, resilience, and well-being in neurology.^17^ The study by Di Liberto et al. adds an interesting observation. Compared with survey respondents who reported disengagement and disinterest, engaged neurologists reported higher professional satisfaction, social support, more advanced career stage, and less burnout.

How do we develop engaged trainees? One hint from the study by Di Liberto et al. may be to focus on flourishing—a state of well-being characterized by positive emotion, engagement, strong relationships, meaning, and achievement.^18^ In some respect, exposure to outpatient neurology—the focus of the first article in this issue—may be a part of that solution. In that study, increasing outpatient exposure was associated with greater patient ownership, less job stress and fatigue, and greater personal reward in the residents' clinic work and their time on inpatient service. Trainees can thrive during high-challenge, high-stress situations.^19^ Helping them to find meaning and fulfillment in or outside of the hospital will position us to address the needs of patients with neurologic disease for years to come. Enjoy this issue of *Neurology: Education*. Happy reading.

## References

[1] Naley MA, Elkind MSV. Outpatient training in neurology: history and future challenges. Neurology. 2006;66(1):E1-E6. doi:10.1212/01.WNL.0000191319.07563.EB. 16401831

[2] Walshe FMR, London FRCP. Training of the neurologist. Arch Neurol Psychiatry. 1933;29(2):368-381. doi:10.1001/ARCHNEURPSYC.1933.02240080158013.

[3] Corboy JR, Boudreau E, Morgenlander JC, Rudnicki S, Coyle PK. Neurology residency training at the millennium. Neurology. 2002;58(10):1454-1460. doi:10.1212/WNL.58.10.1454. 12034779

[4] Pedley TA. Contemporary issue: the changing face of academic neurology: implications for neurologic education at the millennium. Neurology. 1999;53(5):906-914. doi:10.1212/WNL.53.5.906. 10496244

[5] Gelb DJ. Teaching neurology residents in the outpatient setting. Arch Neurol. 1994;51(8):817-820. doi:10.1001/ARCHNEUR.1994.00540200097022. 8042931

[6] Ringel S, Vickrey B, Keran C, Bieber J, Bradley W, Ringel SP. Issues in neurologic practice: training the future neurology workforce. Neurology. 2000;54(2):480-484. doi:10.1212/wnl.54.2.480. 10668718

[7] Roy S, Fu K, Ryan TE, Bordelon Y, Flippen CC, Keener AM. Education research: the impact of an X + Y schedule model on neurology residency training. Neurol Educ*.* 2022;1(2):e200017. doi:10.1212/NE9.0000000000200012. PMC12339223 40809508

[8] O'Rourke P, Tseng E, Chacko K, Shalaby M, Cioletti A, Wright S. A national survey of internal medicine primary care residency program directors. J Gen Intern Med. 2019;34(7):1207. doi:10.1007/S11606-019-04984-X. 30963438 PMC6614222

[9] Osborn R, Bullis E, Fenick AM, Powers E, Banker S, Asnes A. X + Y scheduling in pediatric residency: continuity, handoffs, and trainee experience. Acad Pediatr. 2019;19(5):489-494. doi:10.1016/J.ACAP.2019.05.001. 31077879

[10] Noronha C, Chaudhry S, Chacko K, et al. X + Y scheduling models in internal medicine residency programs: a national survey of program directors' perspectives. Am J Med. 2018;131(1):107-114. doi:10.1016/J.AMJMED.2017.09.012. 28970031

[11] Gleicher ST, Hurd CJ, Weisner PA, Mendelson AM, Creutzfeldt CJ, Taylor BL. Curriculum innovations: implementing a neuropalliative care curriculum for neurology residents. Neurol Educ. 2022;1(2):e200021. doi:10.1212/NE9.0000000000200021. PMC12339220 40809501

[12] Issenberg SB, McGaghie WC, Petrusa ER, Gordon DL, Scalese RJ. Features and uses of high-fidelity medical simulations that lead to effective learning: a BEME systematic review. Med Teach. 2005;27(1):10-28. doi:10.1080/01421590500046924. 16147767

[13] Tchopev ZN, Nelson AE, Hunninghake JC, Cacic K, Cook MK, Jordan MC. Education research: high fidelity simulation of acute neurology enhances rising resident confidence: results from a multicohort. Neurol Educ. 2022;1(2):e200022. doi:10.1212/NE9.0000000000200022. PMC12339216 40809506

[14] Pergakis MB, Chang WTW, Gutierrez CA, et al. Education research: high-fidelity simulation to evaluate diagnostic reasoning reveals failure to detect viral encephalitis in medical trainees. Neurol Educ. 2022;1(2):e200020. doi:10.1212/NE9.0000000000200020. PMC12339219 40809507

[15] Lemus HN, Dworetzky BA, Bubrick EJ, Cosgrove GR, Tobochnik S. Education research: evaluation of epilepsy surgery education in epilepsy and clinical neurophysiology fellowship programs. Neurol Educ. 2022;1(2):e200018. doi:10.1212/NE9.0000000000200018. PMC12339225 40809504

[16] Di Liberto G, Baldizzi G, Carvalho V, et al. Education research: Impact of burnout on neurology residents and research fellows in Europe. Neurol Educ. 2022;1(2):e200035. doi:10.1212/NE9.0000000000200035. PMC12339222 40809503

[17] Busis NA, Shanafelt TD, Keran CM, et al. Burnout, career satisfaction, and well-being among US neurologists in 2016. Neurology. 2017;88(8):797-808. doi:10.1212/WNL.0000000000003640. 28122905 PMC5344080

[18] Slavin SJ, Hatchett L, Chibnall JT, Schindler D, Fendell G. Helping medical students and residents flourish: a path to transform medical education. Acad Med. 2011;86(11):e15. doi:10.1097/ACM.0B013E3182316558. 22030663

[19] Dewey J, Encandela J, Moeller J. Thriving in neurology residency: an appreciative inquiry approach. Neurology. 2022;98(13):E1397-E1405. doi:10.1212/WNL.000000000020003. 35101910

